# Hypertension and associated factors among university students in Gondar, Ethiopia: a cross-sectional study

**DOI:** 10.1186/1471-2458-14-937

**Published:** 2014-09-09

**Authors:** Takele Tadesse, Henok Alemu

**Affiliations:** Institute of Public Health, the University of Gondar, Gondar, Ethiopia; School of Medicine, College of Medicine and Health Sciences, the University of Gondar, Gondar, Ethiopia

**Keywords:** Hypertension, College students, University of Gondar, Ethiopia

## Abstract

**Background:**

Hypertension causes considerable morbidity and mortality worldwide. However, evidences on the burden of hypertension and associated factors are lacking among college students in resource-poor settings. This study measured the prevalence of hypertension and associated factors among university students in Gondar, Ethiopia.

**Methods:**

Institution-based cross-sectional study was conducted among randomly selected college students in Gondar, Ethiopia. Trained data collectors administered a pre-tested and structured WHO STEPS questionnaire for data collection. Data were entered using the EPI INFO version 2002 a statistical soft ware. Stata version 11.0 was also employed for descriptive and logistics regression analysis.

**Results:**

A total of 610 college students were screened for hypertension of which 453 (74.4%) were male and 157(25.6%) female with the male to female sex ratio of 2.9:1. The prevalence of hypertension was 7.7%. Higher rates of hypertension were observed among male [AOR: 3.12, 95% CI (1.16- 8.36)], overweight [AOR: 6.92, 95% CI; (2.65-18.07)] and participants who had sleep duration of ≤5 hours [AOR: 3.48, 95% CI (1.69-7.15)].

**Conclusions:**

A high burden of hypertension was observed among college students in Gondar, Ethiopia. Male sex, overweight and sleep duration of ≤5 hours were identified as independent risk factors for the disease. Preventive measures, such as increasing awareness and early screening for the disease in young adults warranted.

## Background

Hypertension is responsible for an estimated 7.5 million deaths per year worldwide [1]. It doubles the risk of cardiovascular diseases, such as coronary heart diseases, congestive heart failure, stroke, renal failure, and peripheral arterial diseases [2]. Hypertension has been referred to as a “silent killer” [3, 4]. By 2020, studies indicate that mortality by cardiovascular diseases is expected to increase by 120% for women and 137% for men [3]. Hypertension has emerged as an important medical and public health concern in sub-Saharan African countries in addition to the damage being caused by HIV, tuberculosis, and malaria [5]. Hypertension places an excessive financial burden on populations and health care systems by consuming scarce resources [6].

Many factors have been identified as risk factors for hypertension. Socio-demographic factors, such as male gender, advanced age, parental history of hypertension, diabetes mellitus and behavioral factors, like body mass index, sleep duration, smoking, and alcohol consumption are significant predictors of hypertension [7].

Currently Sub-Saharan African countries are experiencing one of the most rapid epidemiological transitions characterized by increasing urbanization and changing lifestyle, which in turn have raised the incidence of non-communicable diseases, especially cardiovascular ailments [5]. The prevalence of overweight and obesity is growing in Sub-Saharan African countries, while the competing epidemics of malnutrition still exists [8–10]. However, studies that clarify the magnitude and risk factors for hypertension among college students are lacking in Ethiopia. Moreover, the country has no national strategy for the prevention and control of chronic diseases or their risk factors. Therefore, this study aimed to measure the prevalence of hypertension and associated factors among college students in Gondar, Ethiopia.

## Methods

A cross-sectional study was conducted to measure the prevalence of hypertension and associated factors in the University of Gondar, Ethiopia, from December 2012 to January 2013. The University of Gondar is found in the historical town of Gondar located 750 km Northwest of Addis Ababa in the North Gondar zone of the Amhara National Regional State. Currently, the total enrolment of the University in the 56 undergraduate and 64 postgraduate programs divisions which consist of regular, extension, distance, summer, and Ph.D programs is 13632, 4269 female and 9363 male.

### Participants and data collection

All regular undergraduate students aged 18 ≥ years of the University of Gondar were the source population. Sample size was calculated on the Open-EPI sample size calculator software using a 5% level of significance, 28.3% prevalence of high blood pressure [11], the total regular undergraduate students of 13632, a 5% margin of error, and a design effect of 2. The final calculated sample size was 610. Four nurses trained for two days on tools and field methods were involved in the data collection processes. The interview questionnaire was structured into three sections (socio-demographic characteristics, behavioral-physical factors, and measurements). The WHO STEPS questionnaire [12] was adopted to collect data on selected behavioral and lifestyle characteristics. The questionnaire was pretested on 10% of the study participants found outside the study area and modifications were made on the basis of the findings. After the interview, participants’ heights and weights were measured and recorded by the interviewers. Weight was measured using an electronic platform model weighing scale to the nearest 0.1 kg. Height was recorded to the nearest 0.1 cm using a microtoise. Weight measuring scales were checked and adjusted at zero level between each measurement, and height was measured following the standard steps. Blood pressure was measured three times in a sitting position using a standard mercury sphygmomanometer BP cuff with the appropriate cuff size that covered two-thirds of the upper arm after the participant had rested for at least five minutes and no smoking or caffeine 30 minutes before measurement. Consecutive measurements were taken five-to-ten minutes after the first measurement. Finally, the average of the last two BP measurements was calculated to determine the BP status of the participant.

### Data management and statistical analysis

Data were entered using the EPI INFO version 2002 statistical software and analyzed using the Stata version 11.0 (Stata, College Station, TX, USA). Descriptive statistics, like frequency distribution, mean, and percentage were employed for most variables. A forward stepwise binary logistic regression analysis was done to assess the relative importance of the explanatory variables on the dependent variable (hypertension). To avoid excess of variables and unstable estimates in the subsequent model, only variables which reached a p-value less than 0.3 were kept in the subsequent analyses. The odds ratio (OR) with a 95% confidence interval (CI) was used to test the statistical significance of variables.

### Ethical considerations

The study protocol was reviewed and approved by the Institutional Review Board of the University of Gondar. Written consent was obtained prior to each interview and interviewee anonymity was guaranteed. Participants who had hypertension by our measurement were referred to nearby health facilities for further diagnosis and treatment.

### Operational definitions

#### Hypertension

A sustained high blood pressure (SBP ≥140 or DBP ≥90 mmHg) [3] or reported regular use of anti-hypertensive medication(s).

#### Pre-hypertension

SBP is 120–139 mmHg or DBP 80–89 mmHg.

#### Normal

SBP is 90–119 mmHg and DBP 60–79 mmHg.

#### Hypotension

SBP is <90 mmHg and DBP <60 mmHg.

Body Mass Index: Calculated as weight in kilograms divided by height in square meters and interpreted as underweight (BMI < 18.5), normal (18.5 - 24.9), overweight (25.0 - 29.9) and obese (≥30.0).

#### Smoking

Light smoker (≤10 cigarettes daily), moderate smoker (≤20 cigarettes daily), and heavy smoker (>20 cigarettes daily).

#### Alcohol drinking

Three or more drinks per day is called “heavy,” and lesser amounts “light,” drinking(30).

### Physical activity level

#### High

Vigorous-intensity activity on at least 3 days achieving a minimum of at least 1,500 MET minutes/week OR 7 or more days of any combination of walking, moderate- or vigorous intensity activities achieving a minimum of at least 3,000 MET-minutes per week.

#### Moderate

Three or more days of vigorous-intensity activity of at least 20 minutes per day or, 5 or more days of moderate-intensity activity or walking of at least 30 minutes per day OR 5 or more days of any combination of walking, moderate- or vigorous intensity activities achieving a minimum of at least 600 MET-minutes per week.

#### Low

A person not meeting any of the above mentioned criteria falls in this category.

## Results

### Characteristics of the study population

A total of 610 college students aged ≥18 years were screened for hypertension. The majority, 453 (74.4%), of the students were male and 157(25.6%) female with the male to female sex ratio of 2.9:1. The mean age of the students was 21 with a standard deviation of 1.82 years. About 20% had family history of chronic diseases. Sixty percent of the participants came from urban areas. Table 1 shows the socio-demographic characteristics of the study population.

**Table 1:** **Distribution of study population by socio-demographic characteristics in Gondar, Ethiopia from December 2012 to January 2013**

Variables	Number	Percent
**Sex**		
Male	453	74.4
Female	157	25.6
**Mean age, years (±SD)**	21 (1.82)	
**Religion**		
Christian	541	89.7
Muslim	51	8.3
Other	18	1.7
**Number of years in college**		
1	173	28.5
2	229	37.5
≥3	208	34.0
**Family history of chronic diseases**		
Hypertension	59	9.7
Kidney disease	21	3.4
Heart disease	27	4.4
Diabetes	16	2.7
None	487	79.8
**Pocket money(birr per month)**		
<200	345	56.6
200-500	194	31.8
>500	71	11.6
**Residence**		
Urban	366	60.0
Rural	244	40.0

### Smoking, khat chewing, and alcohol consumption

Sixteen (2.6%) of the students reported smoking cigarettes of whom 6 (40%) were daily smokers. About 10% reported current khat chewing. Out of the khat chewers, 9.8% of the males chewed khat daily. About 7% of the students took alcohol on daily. The mean (±SD) sleep duration for the students was 6.8 (±1.83) hours (Table 2).

**Table 2:** **Distribution of behavioral risk factors among college students in Gondar, Ethiopia from December 2012 to January 2013**

Variables	Male	Female	Total
**Do you smoke cigarettes?**			
Yes	14(3.1)	2(1.3)	16(2.6)
No	439(96.9)	155(98.7)	594(97.4)
**In a week how many days did you smoke?**			
Daily	5(38.4)	1(50.0)	6(40.0)
1-4 days	4(30.8)	1(50.0)	5(33.3)
4-6 days	4(30.8)	0	4(26.7)
**Have you chewed khat within the past 12 months?**			
Yes	37(16.4)	2(3.0)	39(13.4)
No	189(83.6)	64(97)	253(86.6)
**During the past 12 months, how frequently have you chewed khat?**			
Daily	5(9.8)	0	5(8.8)
5-6 days per week	6(11.8)	0	6(10.5)
1-4 days per week	4(7.8)	1(16.7)	5(28.1)
1-3 days per month	13(25.5)	3(50)	16(43.9)
Less than once a month	23(45.1)	2(33.3)	25(43.9)
**Have you consumed an alcoholic drink within the past 12 months?**			
Yes	192(42.4)	40(27.8)	232(38.0)
No	261(57.6)	117(22.2)	378(62.0)
**During the past 12 months, how frequently have you consumed an alcoholic drink?**			
Daily	9(4.4)	1(2.7)	10(7.1)
5-6 days per week	4(2.0)	0	4(1.7)
1-4 days per week	15(7.4)	2(5.4)	17(7.1)
1-3 days per month	54(36.5)	5(13.5)	59(24.5)
Less than once a month	122(59.8)	29(78.4)	151(62.7)

### Body-mass-index (BMI)

Weight and height measurements were conducted for all individuals. The mean (±SD) BMI was 19.8(±2.38). The majority (59.8%) had a normal BMI, while 3.9% were overweight. The prevalence of obesity was 0.7%; underweight was 35.6%.

### Physical activity level

About 20.2% (23.4% of the males and 10.8% of the females) were classified as having a high (vigorous) physical activity level. Only 14.8% had moderate physical activity level and rest 65.1% had low physical activity level.

### Blood pressure

A total of 47(7.7%) students were hypertensive. About 35.7% and 55.1% had pre-hypertensive and normal blood pressure, respectively. The rest 1.8% had hypertensive blood pressure.

### Multivariate logistic regression analysis

Results from the multivariate analyses using binary logistic regression model showed that male sex, overweight and short sleep duration were found to be independent risk factors for hypertension (Table 3). A higher prevalence of hypertension was observed among male [AOR: 3.12, 95% CI: (1.16-8.36)], overweight [AOR: 6.92, 95% CI: (2.65-18.07)] and participants who had sleep duration of ≤ 5 hours per day [AOR: 3.48, 95% CI: (1.69-7.15)].

**Table 3:** **Bivariate and multivariate analyses of risk factors for hypertension among college students in Gondar, Ethiopia from December 2012 to January 2013**

Characteristics	Yes	No	COR (95% CI)	AOR (95% CI)
**Sex**				
Male	42	412	3.08(1.14-9.02)	3.12(1.16-8.36)^α^
Female	5	151	1.0	1.0
**Body mass index**				
Normal	27	337	1.0	1.0
Underweight	9	209	0.54(0.23-1.22)	0.57(0.26-1.26)
Overweight	11	17	808(3.16-20.53)	6.92(2.65-18.07)^α^
**Sleep duration**				
≤ 5 hours	35	257	3.47(1.70-7.23)	3.48 (1.69-7.15)^α^
> 5 hours	12	306	1.0	1.0

*COR* Curd odds ratio, *AOR* Adjusted odds ratio, *CI* Confidence Interval, ^*α*^Statistically significant, P < 0.05.

## Discussion

This institution based cross-sectional study identified hypertension as a significant health problem among college students aged ≥18 years old. In this study the prevalence of hypertension was 7.4%. This finding is consistent with that of a study conducted in Kuwait which reported that about 7% of students had hypertension [13]. The prevalence of hypertension in this study was lower than that reported in Nigeria (19.3%) [14], Tunisia (35.1%) [15], Gamibia (38.0%) [16] and Ethiopia (28.3%) [11]. The differences may be due to differences in data collection methods, population age groups studied, and time.

Our study showed a positive association between sex and hypertension in which the risk of hypertension increases with being men, which is in line with several studies [13, 15, 17, 18]. It is also important to mention that overweight people were at higher risk of hypertension compared to normal BMI. This finding was in line with previous reports from Tunisia, Portugal, and sub-Saharan African countries. A study done in sub-Saharan countries shows that blood pressure was found to be associated with BMI, and BMI independently predicted blood pressure level in all study participants [5]. A study done in Tunisia showed independent relationships between BMI and hypertension [15].There was a high prevalence of hypertension among overweight (50.8%) in a Portuguese [19].

We also observed that sleeping ≤5 hours per day was significantly associated with hypertension. In the Sleep Heart Health Study, subjects sleeping ≤5 5 hours per day had a higher frequency of hypertension [20] which was in line with this study which found that students who slept ≤ 5 hours per day were about four times at risk to be hypertensive. Compared to a group sleeping 7 hours, individuals sleeping 5 hours or less had an increased risk of hypertension [21] Sleep deprivation was linked to increased prevalence of hypertension. Participants reporting difficulty falling asleep or sleep continuity problems had a slightly increased risk of hypertension at follow-up, even after controlling the confounders [22].

Unlike other studies done so far [23, 24], cigarette smoking, harmful use of alcohol and use of khat were not significantly associated with hypertension in this study. This might be due to the low prevalence of these factors in the community studied. Physical activity level and urban life were significantly associated with the likelihood of hypertension [25–28] in other studies, but we didn’t find any association between the two.

The main limitations of this study were that measurements were taken throughout the day, without taking circadian variations into consideration and that it was limited to young people aged 25 years and below which made comparisons with other studies difficult.

## Conclusions

This study revealed that the prevalence of hypertension was high. Male sex, overweight, and sleeping ≤5 hours per day were identified as independent risk factors for hypertension. Policy makers and program managers need to develop targeted and cost effective intervention programs that will have the greatest impact on hypertension and to increase awareness among university students about hypertension and the need to screen for the disease.
